# Sensory comfort: an overlooked dimension in neuropathic pain trials and management

**DOI:** 10.1097/PR9.0000000000001471

**Published:** 2026-07-06

**Authors:** Louis Tremblais, Anne-Lise Garel, Charles Quesada

**Affiliations:** a Service de Chirurgie de la Main, CHU Edouard Herriot, Hospices Civils de Lyon, France; b Équipe NEUROPAIN Inserm U1028 (CRNL), Université Jean Monnet (Saint-Étienne), France

## Abstract

Sensory comfort represents an underrecognized dimension in neuropathic pain requiring validated assessment and therapeutic strategies addressing somatosensory, psychological, and emotional components.

## 1. Introduction

Neuropathic pain affects 7% to 10% of the global population^2^ and represents a considerable burden for patients.^1^ Current therapeutic approaches focus primarily on pain intensity reduction.^19^ Yet, many patients report persistent discomfort even when pain is controlled,sensations described as “tiring,” “annoying,” or “nagging” but not necessarily painful. These descriptors, familiar from the McGill Pain Questionnaire, reveal a continuum of sensory experiences between symptom absence and frank pain, where nonpainful but distressing sensory symptoms remain underrecognized despite their impact on function and quality of life.^11^

This “sensory discomfort” directly impacts function, return to work, and quality of life, independently of pain intensity. We propose “sensory comfort” as an essential outcome measure in peripheral neuropathic pain management, defined as “*a state of sensory well-being induced by harmonious tactile perception, where stimuli are integrated without causing emotional or cognitive disturbance, allowing automatic and fluid interaction with the environment*.”

The aim of this article was to (1) demonstrate why current approaches miss this essential dimension, (2) define sensory comfort as a patient-centered outcome, (3) propose practical assessment and restoration tools, and (4) call for a paradigm shift in research and clinical practice.

## 2. Why current approaches miss the complete sensory experience

The neuroanatomical organization of peripheral nerves explains the inseparability of somatosensory disorders and neuropathic pain. Nociceptive Aδ and C fibers coexist with tactile Aβ fibers within the same nerve trunk. During peripheral nerve injury, whether traumatic, metabolic, infectious, or iatrogenic, these pathways are simultaneously altered. Yet, clinical focus remains oriented toward pain. Clinical trials use pain intensity as the primary outcome, sometimes supplemented by functional measures (IMMPACT recommendations^22^). Somatosensory disorders, when assessed, are evaluated through quantitative tests that do not capture patients' lived experience. This asymmetry creates a major therapeutic blind spot.

The consequences are tangible, particularly in the hand, whose innervation is exceptionally dense,a digital pulp contains more than 241 receptors per cm^2^, 10 times greater than the foot.^7^ Withdrawal behaviors driven by tactile discomfort rather than pain can progress into movement avoidance and segmentary exclusion syndrome,^21^ directly contradicting somatosensory rehabilitation principles.

In diabetic neuropathies, patients describe “constant discomfort” in the feet impacting gait and balance, independent of pain.^3^ In chemotherapy-induced neuropathies, “non-painful but unbearable” paresthesia limit fine activities and persist after pain resolution.^12^ Sensory discomfort thus constitutes a distinct, clinically relevant dimension.

The neurophysiological basis of this continuum is well established. The gap between nociceptive threshold (∼39°C for cutaneous mechano-heat nociceptors) and pain tolerance (∼45°C) described by Yarnitsky et al.^24^ delineates precisely the sensory territory we address: stimuli that activate nociceptors, register as aversive, yet fall short of frank pain. Chesterton et al. operationalized this distinction, separating pain threshold (first discernible sensation of pain) from the preceding experience of “pressure or discomfort”.^6^ That threshold and tolerance answer to different determinants, one peripheral and physiological, the other contextual and psychological, argues against reducing sensory comfort to a single nociceptive dimension.^23^

## 3. Defining sensory comfort: a patient-centered outcome

The traditional goal of “restoring normal sensation” after peripheral nerve injury is often unrealistic. Age, comorbidities, sleep quality, and emotional state may prevent complete return to preinjury state, resulting in perceived therapeutic failure.

The concept of sensory comfort proposes a paradigm shift: our goal is not “normal” but “comfortable” sensation. This recognizes that a new sensory normality can be functional, provided it is comfortable for the patient.

We conceptualize sensory comfort along two interdependent dimensions. This binary structure mirrors the foundational IASP definition of pain as “an unpleasant sensory and emotional experience”^17^ and is supported by neuroimaging evidence of anatomically distinct somatosensory and affective–cognitive circuits.^5^

First, neurophysiological and somatosensory comfort encompasses the full continuum of peripheral sensory experience, from nonpainful but aversive tactile input to frank nociception, reflecting the covulnerability of Aδ, C, and Aβ fibers and the threshold/tolerance dissociation detailed above. In clinical terms, somatosensory comfort requires sufficient tactile and proprioceptive function to interact with the environment without discomfort, enabling the dexterity and sensory-guided motor control on which upper limb function depends. It should be noted that frank pain and somatosensory comfort are rarely reconcilable in clinical reality, which is precisely why addressing nociception alone, without attending to the broader sensory experience, leaves so many patients incompletely treated.^10^

Second, psychoemotional comfort addresses the cognitive and affective burden of living with persistent sensory discomfort.^8,14^ Illness beliefs, confidence in recovery, and illness perceptions shape the subjective experience of sensory symptoms at least as much as peripheral input does. For many patients, sensory discomfort becomes entangled with worry and kinesiophobia, particularly in traumatic contexts where touch may reactivate emotional distress, a re-experiencing phenomenon well-documented in posttraumatic stress disorder.^4^ These emotional responses, if unaddressed, may contribute to chronification: Recent mediation analyses show that emotional distress, fatigue, and cognitive complaints are strong predictors of disability in chronic pain, sometimes more than pain intensity itself.^20^

This paradigm shift, from “return to normal” toward “functional comfort”, mirrors evolutions in oncology (from survival to quality of life) and mental health (from symptom reduction to social functioning). It is overdue in neuropathic pain management.

## 4. Assessing and restoring sensory comfort: practical tools

### 4.1. Changing our clinical question

Rather than “Are you in pain?”, we propose: “Is your hand (your foot, your affected area) comfortable?” By emphasizing comfort, this question draws on positive communication models known to influence pain perception and patient experience,^15^ and creates space to discuss symptoms that lie between symptom absence and frank pain,experiences that clinicians may reductively label as “pain” absent more nuanced terminology. Legitimizing and naming this experience is an essential first therapeutic step.

### 4.2. Assessment tool

**Figure 1.**
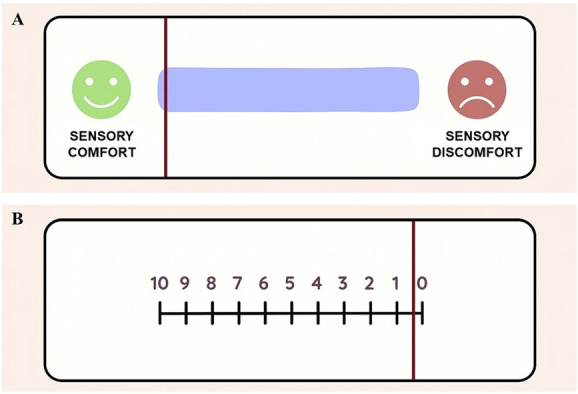
Scale for the assessment of sensory discomfort: The front side (A) is accessible exclusively to the patient, whereas the reverse side (B) is visible only to the practitioner. The red line represents a movable marker manipulated by the patient to indicate the perceived level of discomfort, with 0 representing comfortable normal state (as before injury) and 10 representing discomfort so intense it becomes painful.

We use a visual analog scale for sensory discomfort assessment (Fig. 1). The patient moves a cursor between 0 (“comfortable, as before injury”) and 10 (“discomfort so intense it becomes painful”). This scale is currently undergoing psychometric validation at our center and is also used in nursing injection care^25^ and postanesthesia care units.^9^ Developing and validating standardized instruments across populations and cultures, capturing both neurophysiological/somatosensory and psychoemotional dimensions, remains an urgent priority.

### 4.3. Therapeutic strategies

**Figure 2.**
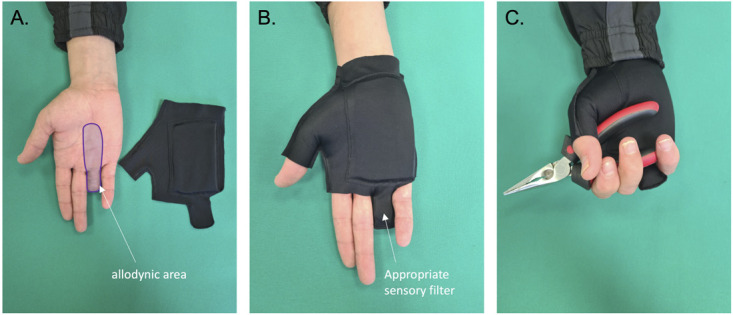
Sensory filter as a rehabilitation tool for mechanical allodynia. (A) Palmar view of the patient's hand showing the allodynic area (purple outline) localized over the thumb and thenar eminence, alongside the custom-made Lycra sensory filter device. (B) The appropriately fitted sensory filter in place, covering the allodynic area and providing controlled mechanical input to the sensitized skin territory. (C) With the sensory filter applied, the patient is able to perform forceful and dexterous manual tasks, here gripping pliers, that would be otherwise impossible due to contact-evoked pain in the uncovered allodynic zone.

Physical interventions: We have developed the concept of a “sensory filter,” a personalized interface between skin and environment aimed at restoring comfort. For a patient with uncomfortable touch-provoked hyperesthesia, a custom-made Lycra glove with neoprene or silicone additions (Fig. 2) can absorb mechanical stress and restore functional capacity. By restoring comfort, this sensory filter allows patients to use their hand again, multiplying somatosensory interactions and promoting natural self-rehabilitation.

Other validated physical interventions include graded sensory desensitization,^16^ an approach aimed at somatosensory rehabilitation in patients presenting with hyperesthesia, progressively recalibrating altered tactile perception through controlled stimulation hierarchies, such as texture-graded sensory retraining or vibrotactile stimulation, and always within the patient's comfort tolerance.

Psychological and psychophysiological interventions: Pain neuroscience education helps patients understand neuropathic mechanisms,^13^ reducing catastrophizing and kinesiophobia. Acceptance and Commitment Therapy and mindfulness approaches have demonstrated efficacy in chronic neuropathic pain.^14^ Combining these with physical rehabilitation creates a multidimensional framework targeting both nociceptive and nonnociceptive sensory dimensions.

A concrete implementation pathway for these strategies in clinical neuropathic pain services would involve three sequential steps: (1) systematic screening using the sensory comfort VAS at each consultation, alongside standard pain intensity scales; (2) stratification of patients based on their dominant comfort deficit (nociceptive, somatosensory, psychoemotional) to guide individualized treatment selection; and (3) iterative reassessment to monitor comfort trajectory and adjust interventions accordingly. This structured approach would enable prospective data collection to build the evidence base required for guideline development.

## 5. Call for a paradigm shift

Research priorities include the following: developing a core outcome set explicitly incorporating sensory comfort via international consensus following the IMMPACT framework, validating assessment scales across etiologies and cultures, and systematically including sensory comfort measures in therapeutic trials to dissociate effects on pain and comfort.

Implementation science demonstrates that integrating sensory dimensions into routine care requires structured strategies and dedicated training.^18^ Clinicians should routinely assess sensory comfort alongside pain, validate patients' sensory experiences, and address cognitive-behavioral barriers to recovery. Evidence-based interventions targeting sensory comfort restoration need development, and this construct warrants inclusion in professional curricula.

## 6. Conclusion

Peripheral nerves are primarily sensory organs whose integrity conditions our daily environmental interaction. Sensory comfort is not a luxury or secondary objective, but a necessity for functional recovery, psychological well-being, and therapeutic success.

The tools to assess and restore this comfort already exist in embryonic form within our practices. What we lack is their rigorous validation, systematic dissemination, and above all, their recognition as a central care dimension.

We call on researchers to include sensory comfort in their protocols, clinicians to ask patients about comfort and make it a primary objective, patient associations to advocate for this recognition, and funders to support the development of necessary tools.

## Disclosures

The authors have no conflict of interest to declare.
